# Features of interactive recipes that support college students’ self-efficacy in preparing produce

**DOI:** 10.3389/fpubh.2025.1659190

**Published:** 2025-12-02

**Authors:** Kim Spaccarotella, Sofiia Korotka

**Affiliations:** 1 Department of Biological Sciences, Kean University, Union, NJ, United States; 2 Department of Integrative Sciences and Technology, Kean University, Union, NJ, United States

**Keywords:** produce, recipe book, self-efficacy, college students, nutrition

## Abstract

**Introduction:**

College students often struggle with produce intake due to factors including limited kitchen access, lack of culinary skills, nutrition knowledge, time, and money. Previous research identified recipe videos as useful for improving college students’ self-efficacy in the kitchen and in preparing healthy but less-familiar food items including fresh produce provided by a campus food pantry or garden. The purpose of the current project was to develop and test an interactive, student-friendly recipe book and gather feedback about additional resources that may help students increase self-efficacy in preparing produce.

**Methods:**

The Cognitive Theory of Multimedia Learning was used to create brief cooking videos for a recipe book featuring common ingredients from a campus garden and food pantry. A short survey assessed nutrition self-efficacy and gathered feedback about the book and resources students felt would be most helpful.

**Results:**

Average daily vegetable intake was 2.05 ± 1.53 servings per day (*n* = 20). Mean self-efficacy in preparing produce shown on the book’s cover was 5.10 ± 1.37 before vs. 5.86 ± 0.864 after viewing the book (*p* = 0.104, effect size 0.190, *n* = 14). Students believed the interactive book was helpful and recommended both web-based and printed materials as future resources.

**Discussion:**

Interactive recipe books may help college students use produce, and feedback from this study can help inform development of education materials for public health nutrition interventions for this population. Research should explore long-term impact of the recipe book across a larger population and with other interactive tools that may support produce consumption.

## Introduction

Low intake of produce is a common and important public health issue among college students. The American College Health Association reported that 28.2% of students consumed an average of three or more servings of vegetables per day, and 17.9% consumed an average of three or more servings of fruit per day (1). However, the Dietary Guidelines recommend consumption of 2.5 cups of vegetables and 2 cups of fruit per day, since both provide many nutrients and may reduce risk of chronic disease (2, 3). Food insecurity is another important public health issue among students that can contribute to low intake of produce. According to the United States Department of Education’s National Postsecondary Student Aid Study, an estimated 23% of college students experienced food insecurity in 2020 (4); further research will be needed to clarify long-term impacts of corona virus disease 2019 (COVID-19) on diet quality and access to fruits and vegetables. College students face additional, unique obstacles in preparing and consuming produce and maintaining other healthy eating habits, including busy schedules that limit meal preparation, lack of cooking skills, knowledge or confidence in food preparation, limited resources, and a reliance on convenience foods, as well as environmental factors such as lack of access to grocery stores and fresh produce (5–9).

With approximately 25 million students enrolled across more than 5,500 postsecondary institutions in the United States during the 2022–23 academic year (10), campuses serve as critical settings for public health nutrition interventions. Although barriers to vegetable consumption pose significant challenges, research has identified several supports that encourage healthy cooking among young adults and can be incorporated into nutrition interventions. For example, increasing access to produce through campus food pantries or gardens may reduce costs and make fruits and vegetables easier to obtain (11). In addition, resources to simplify cooking may help students use the produce they receive. For instance, a focus group with 239 students at Abilene Christian and Baylor Universities found that students desired healthy foods and were interested in cooking to learn more about the composition of ingredients and the preparation methods used to make the meals they consumed (12). Lack of time was a barrier, and providing students with shopping lists, recipes, nutrition information and quick preparation tips was recommended as helpful (12). Another study of 16 young adults pilot tested the effectiveness of short videos on addressing challenges to cooking vegetables and found that the videos decreased student perceptions of time, cost and lack of cooking skills as barriers (7). Moreover, participants appreciated the inclusion of nutrition information about the health benefits of vegetables and desired additional information on macronutrients (7). Among college students, cooking videos may be effective in increasing produce consumption (13, 14) and fruit and vegetable knowledge (15), as well as confidence in cooking at home (6), using fruits and vegetables (14) and preparing lettuce (16). Additional factors that facilitate produce consumption among students include providing recipes that are simple and accessible, quick to make with limited kitchen equipment and easy to follow (5–7, 16). Recipes for fruits and vegetables should also be affordable and use familiar ingredients so that they are practical and relevant to college students in a wide range of communities (8, 13) while helping address their nutrition needs. Although research has identified these features that may help cooks increase confidence in preparing vegetables, there is little, if any, research about how they could function together in a recipe book, which is a common format for presenting recipes, and which other features are most important to include in interactive recipe materials and systems.

Several public health models have been suggested as guides for research in this area. The Social Cognitive Theory has been used to guide development of cooking videos that help students observe and model specific skills needed to successfully prepare produce (14). The Cognitive Theory of Multimedia Learning, which focuses on reducing cognitive load through effective presentation of visual and auditory information in a brief, interactive format to enhance knowledge retention, supports this (17–19). However, although much research exists on tailoring recipes for target audiences and nutrition interventions involving video technology, gaps remain in accessible resources that specifically address college students’ unique characteristics, such as limited cooking skills, familiarity with technology and reliance on commonly available pantry ingredients. Previous research in this area has paired videos with recipes in several formats, including digital recipe cards and written recipes (6, 8). Booklets are another popular form in which recipes are published; however, it is unclear whether students would find these helpful. Likewise, although games and nutrition practice activities helped adolescents increase produce consumption (20), it is unclear whether college students would also find these appealing and perceive them as beneficial as part of interactive recipe materials. Prior research has also found that a QR code for a cooking video and further nutrition information added to packaging of produce harvested from a campus garden favorably improved self-efficacy in preparing produce (16). Thus, the purpose of the current project was to pilot test a series of brief cooking videos paired with a recipe booklet developed for college students to understand whether these increase nutrition self-efficacy and to obtain feedback on features, formats and other resources that college students prefer for produce recipes and nutrition education related to preparing produce.

## Materials and methods

MyPlate Kitchen – the U.S. Department of Agriculture’s website featuring low-cost recipes – and Google, which was previously identified by food pantry clients as a go-to recipe source (21, 22) were used to identify recipes that met or could be modified to meet the USDA’s Dietary Guidelines for Americans (2) and could also be adapted for a smaller yield using ingredients available through the campus food pantry and garden or a nearby supermarket. Selection criteria for recipes were based on previous research (16) and included preparation time under 30 min and use of appliances available to students. These criteria were used to ensure that the recipes were quick, practical, and tailored to the limited resources of college students living on campus with some access to a communal kitchen (8, 23). Six recipes meeting these criteria were selected with input from students to ensure their acceptability for the target audience (Fruit and Banana “Split,” Scrambled Eggs in a Mug, Blueberry Peach Salad, Spinach Salad, Chicken with Green Beans and Microwave Eggplant). The six recipes were chosen to ensure that options for breakfast, lunch and dinner were included and to help students incorporate produce across the day and increase dietary variety, as recommended by the United States Department of Agriculture (2). In addition, previous research pilot-testing recipes with students and young adults used a similar number (6, 8).

To ensure clarity and consistency in the videos, a detailed script was created for each recipe. The script included an introduction, which was designed to engage the audience, a list of ingredients and utensils, step-by-step preparation instructions, which used concise language and helpful tips to ensure successful recipe preparation, serving suggestions and alternatives for substituting ingredients in the recipe (8), and a conclusion highlighting health benefits of the ingredients used (7, 8).

To ensure relevance to a student audience, the videos were recorded by students in a kitchen setting where the speaker could make good eye contact and use a conversational tone to engage with the audience as has been done previously (13, 17). They also showed close-ups of preparation steps similar to cooking videos seen on YouTube. Information was delivered at a moderate-to-fast pace, which has been found to be more engaging (13, 17), and mean length of the videos was 4.81 ± 2.58 min so that students would not lose interest (17). By adhering to the Cognitive Theory of Multimedia Learning, the videos aimed to enhance learning retention and viewer engagement while encouraging practical application of the recipes. The combination of concise content, engaging delivery, and user-friendly presentation was designed to maximize both the educational value and viewer interest (13).

The videos were incorporated into a recipe book. Each page provided a descriptive recipe name and an image of the final product, providing users with a visual reference of the expected results (8, 24). Each page also included an estimated time the recipe would take to prepare, along with a link to the recipe video. Below the video link was a list of ingredients tailored for a small yield and an easy-to-follow set of instructions with nutrient information. The cover of the recipe book included pictures of each dish shown in the book.

To evaluate the effectiveness of the recipe book and videos, a short (approximately 10 min), anonymous questionnaire was used. To ensure students from a wide variety of majors and backgrounds were given the opportunity to respond, the questionnaire was disseminated to the Kean University student body, including undergraduate and graduate students, via email blast with an invitation to participate. A follow-up invitation was sent to science students, as well. The survey was distributed between February and March of 2025. Students had until May of 2025 to provide responses. The questionnaire included sections asking about usual vegetable consumption, adapted from the Short Healthy Eating Index Survey (25), and self-efficacy in preparing produce, which were based on the Cooking and Food Provisioning Action Scale (26). Both measures have been previously validated for use with adults (25, 26). To reduce bias, the survey question about vegetable intake included an explanation to clarify that 1 serving of vegetables was equal to 1 cup of raw vegetables, ½ cup of cooked vegetables, 1 cup of salad or ½ cup of 100% vegetable juice. A picture of a baseball and computer mouse were shown to illustrate 1 and ½ cup serving sizes, respectively. Data on fruit consumption in this population have been reported previously (16). The questionnaire also provided a link to the recipe book, asked students for their thoughts after reviewing the recipe book and at least one video, and asked for examples of other types of nutrition resources (recipe apps, websites with recipes, printed recipe sheets, books or cards, and web games or activities about nutrition and cooking topics like portion size) they thought would help students as part of an interactive website to support the recipe book. To access the videos, students were required to click on a link for the book, which opened as a PDF, choose a recipe, and click on a link within the recipe to view the video. Students were asked to indicate which video they had viewed. The study involving human participants was reviewed and approved as exempt by the Kean University ethics committee, and all participants gave written informed consent before participating. Data were analyzed using IBM SPSS Statistics 30 (IBM, Armonk, NY, USA), and all values are given as means ± standard deviations unless otherwise noted. The qualitative data were reviewed to identify common themes, which were summarized and compared to previous research in this field.

## Results

Twenty students provided data about their usual intake of vegetables, confidence in preparing produce and preparing the produce shown on the recipe book’s cover and use of the campus food pantry. Sixteen students evaluated the recipe book’s helpfulness, and fourteen indicated their confidence in preparing the produce on the recipe book’s cover after viewing the book. Five students indicated previous use of the campus food pantry. Students consumed an average of 2.05 ± 1.53 servings of vegetables per day (Table 1), and 51% consumed fewer than 2 servings per day, which is below recommendations for vegetable consumption (2). The self-efficacy items were scored on a 7-point scale (ranging from 1, “strongly disagree” to 7, “strongly agree”). In general, when preparing produce, students somewhat agreed that they were confident in dealing with unexpected results (5.05 ± 1.24), felt it was easy to accomplish their desired results (5.57 ± 1.21) and solve problems (5.19 ± 1.33) and felt comfortable preparing produce 5.57 ± 1.47 (Table 1). Analysis of variance was used to compare nutrition self-efficacy before and after viewing the recipe book and video. Before viewing the recipe book and video, mean confidence in preparing the produce shown on the cover of the recipe book was 5.10 ± 1.37, and afterwards, mean confidence was 5.86 ± 0.864 (*p* = 0.104, effect size = 0.190, large effect) (27). When asked to evaluate the perceived usefulness of the recipe book using a scale of 1, not helpful to 5, extremely helpful in helping students select and prepare produce from the campus food pantry, students rated the book at a mean of 3.87 ± 0.640, indicating they felt it would be helpful.

**Table 1:** Mean vegetable consumption, self-efficacy in preparing produce and perceived helpfulness of the recipe book.

Variable	Mean ± SD	95% CI
Vegetable consumption (*n* = 20)		
Average vegetable servings per day	2.05 ± 1.53	1.35–2.75
Self-efficacy in preparing produce (*n* = 20)		
Confident in dealing with unexpected results	5.05 ± 1.24	4.48–5.61
Easily accomplish desired results	5.57 ± 1.21	5.02–6.12
Solve problems that arise when preparing produce	5.19 ± 1.32	4.59–5.79
Comfortable preparing produce	5.57 ± 1.47	4.90–6.24
Self-efficacy in preparing produce featured in the recipe book (*n* = 14)		
Comfortable preparing the produce shown on the recipe book’s cover	5.10 ± 1.37	4.46–5.74
Comfortable preparing the produce shown on the recipe book’s cover after viewing the recipe book and video*	5.86 ± 0.864	5.36–6.36
Perceived helpfulness of the recipe book (*n* = 16)		
Usefulness of the recipe book in helping students select and prepare produce from the food pantry	3.87 ± 0.640	3.51–4.22

Response options for the self-efficacy questions ranged from 1 (strongly disagree) to 7 (strongly agree); SD, standard deviation; CI, confidence interval. *p = 0.104, effect size = 0.190. Data were collected from February to March of 2025.

Ten students shared qualitative feedback about the video they watched to help identify features of videos for interactive recipe books that they perceived as most beneficial. For example, several reported liking “how the camera focused on making the meal” or “how the video was shot and shown” and felt this made the process easy to follow for visual learners. They also appreciated that the videos were “easy to follow” and that the recipes “were easy to make” and helpful “especially for newer chefs,” and another student liked the substitutions provided in each video for those who did not have all of the ingredients on hand. Suggestions for improvement included providing a wider variety of salad recipes to “complement more things” and additional pictures of the finished product. Participants felt that imitating cooking videos seen on YouTube and including appetizing pictures of the finished product helped motivate students to try the recipes. However, one participant thought that portraying students in the video made it seem “more like a mandatory school project” compared to a typical cooking video. When asked to comment on other types of nutrition resources that would help students prepare produce, 12 students suggested websites with recipes would be helpful, 9 students suggested recipe apps, 9 suggested printed materials (books, cards or sheets), and 5 suggested web games or activities about portion size and other nutrition or cooking topics.

## Discussion

The current project found that an interactive recipe book with brief cooking videos would help students select and prepare produce from a campus food pantry. In addition, their recommendations provide direction for further development of interactive recipe books and related nutrition education resources for this population.

Similar to previous research, despite the reduced risks of chronic health conditions that are linked with fruit and vegetable consumption (2), many of the students consumed few servings of vegetables (1). This included those who reported using the campus food pantry. The Dietary Guidelines recommend consuming 2.5 servings of vegetables daily (2). This suggests that strategies to increase produce consumption could be beneficial for this population. Several students commented on the helpfulness of the visual presentation and format. This aligns with findings from Roy et al. (8), who noted that including an eye-catching photograph of the dish is a key feature of recipe cards and that use of a bird’s-eye view using a camera angle showing the food from the perspective of the cook was perceived as more helpful in cooking videos. In addition, feedback suggested styling the videos so that they resemble those commonly posted on social media. YouTube has been cited in previous research as a resource for learning about new recipes and ingredients (8). Considering that YouTube is one of the predominant social media platforms used by young adults (18 to 29 years old) (28), this finding suggests that aligning the videos’ format to those in popular online sources may resonate well with students and enhance engagement and accessibility. Furthermore, students appreciated the ability to easily modify the recipes. Including options for substituting ingredients supports students receiving produce from a campus food pantry or garden where the selection may vary by week or season and those who have limited access to supermarkets. Helping students overcome these barriers may improve their ability to incorporate a more varied selection of fruits, vegetables and other ingredients. Participants also stressed the importance of recipes that were easy to follow, which reduces cognitive load (17) and may enhance motivation to cook (8). Similar findings were reported previously with recipe cards and videos (5, 8), suggesting that clear instructions and simple ingredients are important details to consider when developing and presenting recipe books for this population.

Aside from feedback on the videos, participants also expressed interest in other nutrition resources to help them select and prepare produce. One of the popular choices was recipe applications, and previous studies have demonstrated the effectiveness of mobile apps to increase produce consumption. For example, among community food pantry clients, a pilot test of a mobile app called VeggieBooks, which offered 260 vegetable-based recipes with food tips and allowed users to print personalized cookbooks, reported increases in using a wide range of vegetables compared to clients at control pantries not using the app (29). In a study of Australian adults, an app called VegEze significantly increased intake of vegetables by half a serving (30). Future research could explore the development of a recipe app tailored to college students and campus food pantry clients while integrating successful features, such as customizable content.

Students also felt that websites with recipes, games and activities about nutrition and cooking topics would be helpful. MyPlate Kitchen and other U.S. Department of Agriculture resources can be used to develop evidence-based materials tailored to college students that incorporate regional flavors and ingredients and encourage students to customize their food choices using MyPlate (21). This would allow students and campus food pantry clients at multiple institutions to benefit from the education materials and adapt them to their needs. In the current project, the recipe book was saved as a PDF so that it could be viewed onscreen or easily printed, yet nine students expressed a desire for printed recipe sheets, books or cards in addition to electronic resources. Similarly, when Clarke et al. (29) surveyed community food pantry clients, participants desired both printed recipes and the option to view recipe content on their phone’s screen. Further supporting the importance of visual guidance, Surgenor et al. (18) reported that participants felt using a cooking video with a printed recipe improved their self-efficacy and enjoyment of cooking compared to using a printed recipe alone, suggesting that integrating digital tools can further support recipe engagement. These concepts were incorporated into the design of the current interactive recipe book. To keep the recipe videos accessible, the recipe book can be redesigned into printable recipe cards with QR codes instead of hyperlinks. This approach, supported by previous research (16), allows users to scan QR codes on the printed material they need and instantly access the corresponding cooking videos, maintaining interactive learning while catering to varied format preferences.

Students also expressed interest in additional nutrition resources such as web games or activities about nutrition and cooking topics like portion size. This aligns with prior research that has shown such activities reinforce learning and make nutrition education enjoyable. For example, among low-income adults in the Food eTalk program (31), participants felt interactive elements, such as self-quizzes and games, would improve engagement, which may reinforce learning and make nutrition education more enjoyable. Similarly, a systematic review of food literacy interventions among youth and young adults using games and other technology, such as web-based platforms, reported positive changes in produce intake (20). Additional research should explore the use of these resources with college students and campus food pantry clients.

This study has several limitations. Participants were recruited from one institution, which limits the sample size and generalizability of the findings. Expanding the scale of future studies to other college campuses could help refine the recipe book’s applicability across a wider student population and increase the number of participants. In addition, institutional security policies required students to sign into their school email to view shared documents like the recipe book, and this may have discouraged some participants from viewing the recipe book and finishing the survey. For the future, the recipe book will be shared on a publicly available project website. Further, to minimize respondent burden, students were only asked to review one video; in the future, a longer project, in which students review multiple videos over a longer time or try preparing the recipes, could provide further insights into preferred features and enable comparisons between multiple types of videos (e.g., comparisons in length, style, etc) and could also assess whether long-term changes in produce consumption or self-efficacy occur following several weeks of intervention materials (15, 29). The current study compared participants’ self-efficacy in preparing vegetables before and after viewing the recipes. A larger intervention could also include a separate group to serve as a control for other factors that may affect self-efficacy and participants’ responses to viewing the recipes and videos. To reduce respondent burden, the current project used self-reported intake data, which can be subject to over and underreporting errors (25). Although biomarker assessments may provide more objective data to confirm intake, they can be invasive, costly and time-consuming (25). Future research with a larger sample should explore which assessment methods provide the most accurate data but are feasible for research among campus pantry clients. Finally, future studies could provide additional follow-up reminders to encourage survey participation and increase the response rate.

This pilot study examined the feasibility of using an interactive recipe book and theory-based videos to support college students with limited access to kitchen resources, simple culinary skills, time constraints, and campus food pantry ingredients. Food insecurity and low vegetable intake among college students have been recognized as a significant public health concern, and campuses frequently open food pantries and start gardens to provide students with produce. However, research about the types of materials that best support students so that they are confident in preparing the produce they receive is limited. Given recent funding cuts to the Supplemental Nutrition Assistance Program’s education component (SNAP-Ed) (32), these findings provide insight to support the development of nutrition education materials that fill the void left in the absence of SNAP-Ed.

Our findings suggest that recipe books with videos using appealing visuals, bird’s-eye angles, clear instructions and ingredient substitutions tailored to college students are perceived as helpful in preparing produce. Additionally, aligning video formats with those in popular online sources, such as YouTube, may resonate well with the audience, enhancing engagement and accessibility. Future research should explore the long-term impact of recipe books and educational videos on college students’ dietary habits and health outcomes. Moreover, our findings provide insight into types of nutrition resources that college students may prefer, which could help campus food pantries, farms and wellness programs focus efforts when designing education materials to support student consumption of produce. Further research should also explore other interactive tools, such as mobile apps, web-based games, online curricula and recipe websites that could provide real-time support for produce and preparation to determine how these may enhance student engagement with nutrition education, strengthen college students’ self-efficacy in preparing produce, promote long-term healthy eating behaviors, and improve accessibility to practical solutions tailored to the needs of college students.

## Data Availability

The datasets presented in this article are not readily available because of institutional policies regarding the privacy of human subjects. Requests to access the datasets should be directed to kspaccar@kean.edu.
